# Tropical forest lianas have greater non-structural carbohydrate concentrations in the stem xylem than trees

**DOI:** 10.1093/treephys/tpad096

**Published:** 2023-08-16

**Authors:** Caroline Signori-Müller, David Galbraith, Julia V Tavares, Simone M Reis, Francisco C Diniz, Martin Gilpin, Beatriz S Marimon, Geertje M F van der Heijden, Camila Borges, Bruno B L Cintra, Sarah Mião, Paulo S Morandi, Alex Nina, Carlos A Salas Yupayccana, Manuel J Marca Zevallos, Eric G Cosio, Ben H Marimon Junior, Abel M Mendoza, Oliver Phillips, Norma Salinas, Rodolfo Vasquez, Maurizio Mencuccini, Rafael S Oliveira

**Affiliations:** Faculty of Environment, Science and Economy, University of Exeter, Exeter EX4 4QE, UK; Department of Plant Biology, Programa de Pós Graduação em Biologia Vegetal, Institute of Biology, University of Campinas, Campinas 13083-862, Brazil; School of Geography, University of Leeds, Leeds LS2 9JT, UK; School of Geography, University of Leeds, Leeds LS2 9JT, UK; School of Geography, University of Leeds, Leeds LS2 9JT, UK; Department of Ecology and Genetics, Uppsala University, Uppsala 756 51, Sweden; Programa de Pós-Graduação da Rede de Biodiversidade e Biotecnologia da Amazônia Legal (BIONORTE), UFAM–UNEMAT, Nova Xavantina 78690-000, Brazil; Laboratório de Ecologia Vegetal, Universidade do Estado de Mato Grosso, Nova Xavantina 78690-000, Brazil; School of Geography and the Environment, Environmental Change Institute, University of Oxford, Oxford OX1 3QY, UK; School of Geography, University of Leeds, Leeds LS2 9JT, UK; School of Geography, University of Leeds, Leeds LS2 9JT, UK; Programa de Pós-Graduação da Rede de Biodiversidade e Biotecnologia da Amazônia Legal (BIONORTE), UFAM–UNEMAT, Nova Xavantina 78690-000, Brazil; Laboratório de Ecologia Vegetal, Universidade do Estado de Mato Grosso, Nova Xavantina 78690-000, Brazil; School of Geography, University of Nottingham, Nottingham NG7 2RD, UK; Programa de Pós-Graduação da Rede de Biodiversidade e Biotecnologia da Amazônia Legal (BIONORTE), UFAM–UNEMAT, Nova Xavantina 78690-000, Brazil; Laboratório de Ecologia Vegetal, Universidade do Estado de Mato Grosso, Nova Xavantina 78690-000, Brazil; School of Geography, University of Leeds, Leeds LS2 9JT, UK; School of Geography, Earth and Environmental Sciences, University of Birmingham, Birmingham B15 2TT, UK; Department of Plant Biology, Programa de Pós Graduação em Biologia Vegetal, Institute of Biology, University of Campinas, Campinas 13083-862, Brazil; Programa de Pós-Graduação da Rede de Biodiversidade e Biotecnologia da Amazônia Legal (BIONORTE), UFAM–UNEMAT, Nova Xavantina 78690-000, Brazil; Laboratório de Ecologia Vegetal, Universidade do Estado de Mato Grosso, Nova Xavantina 78690-000, Brazil; Instituto de Ciencias de la Naturaleza, Territorio y Energías Renovables, Pontificia Universidad Católica del Perú, Lima 15088, Peru; Instituto de Ciencias de la Naturaleza, Territorio y Energías Renovables, Pontificia Universidad Católica del Perú, Lima 15088, Peru; Instituto de Ciencias de la Naturaleza, Territorio y Energías Renovables, Pontificia Universidad Católica del Perú, Lima 15088, Peru; Facultad Ciencias Biologicas, Universidad Nacional de San Antonio Abad del Cusco, Cusco 08003, Peru; Instituto de Ciencias de la Naturaleza, Territorio y Energías Renovables, Pontificia Universidad Católica del Perú, Lima 15088, Peru; Programa de Pós-Graduação da Rede de Biodiversidade e Biotecnologia da Amazônia Legal (BIONORTE), UFAM–UNEMAT, Nova Xavantina 78690-000, Brazil; Laboratório de Ecologia Vegetal, Universidade do Estado de Mato Grosso, Nova Xavantina 78690-000, Brazil; Facultad Ciencias Biologicas, Universidad Nacional de San Antonio Abad del Cusco, Cusco 08003, Peru; Jardín Botánico de Missouri, Cusco 19231, Peru; School of Geography, University of Leeds, Leeds LS2 9JT, UK; School of Geography and the Environment, Environmental Change Institute, University of Oxford, Oxford OX1 3QY, UK; Instituto de Ciencias de la Naturaleza, Territorio y Energías Renovables, Pontificia Universidad Católica del Perú, Lima 15088, Peru; Jardín Botánico de Missouri, Cusco 19231, Peru; CREAF, Campus UAB, Cerdanyola del Vallés 08193, Spain; ICREA, Barcelona 08010, Spain; Department of Plant Biology, Institute of Biology, University of Campinas, Campinas 13083-862, Brazil

**Keywords:** host tree, liana infestation, soluble sugars, starch

## Abstract

Lianas (woody vines) are important components of tropical forests and are known to compete with host trees for resources, decrease tree growth and increase tree mortality. Given the observed increases in liana abundance in some forests and their impacts on forest function, an integrated understanding of carbon dynamics of lianas and liana-infested trees is critical for improved prediction of tropical forest responses to climate change. Non-structural carbohydrates (NSC) are the main substrate for plant metabolism (e.g. growth, respiration), and have been implicated in enabling tree survival under environmental stress, but little is known of how they vary among life-forms or of how liana infestation impacts host tree NSC. We quantified stem xylem total NSC concentrations and its fractions (starch and soluble sugars) in trees without liana infestation, trees with ˃50% of the canopy covered by lianas, and the lianas infesting those trees. We hypothesized that (i) liana infestation depletes NSC storage in host trees by reducing carbon assimilation due to competition for resources; (ii) trees and lianas, which greatly differ in functional traits related to water transport and carbon uptake, would also have large differences in NSC storage. As water availability has a significant role in NSC dynamics of Amazonian tree species, we tested these hypotheses within a moist site in western Amazonia and a drier site in southern Amazonia. We did not find any difference in NSC, starch or soluble sugar concentrations between infested and non-infested trees, in either site. This result suggests that negative liana impact on trees may be mediated through mechanisms other than depletion of host tree NSC concentrations. We found lianas have higher stem NSC and starch than trees in both sites. The consistent differences in starch concentrations, a long-term NSC reserve, between life forms across sites reflect differences in lianas and trees carbon gain and use. Soluble sugar concentrations were higher in lianas than in trees in the moist site but indistinguishable between life forms in the dry site. The lack of difference in soluble sugars between trees and lianas in the dry site emphasizes the importance of this NSC fraction for the metabolism of plants occurring in water limited environments.

Abstracts in Portuguese and Spanish are available in the supplementary material.

## Introduction

Lianas (woody vines) are important components in forests worldwide, especially lowland tropical forests (Gentry 1991) where they constitute up to 40% of woody stems and up to 35% of woody species richness (Schnitzer and Bongers 2011). Liana abundance and biomass are increasing throughout the Neotropics, with potentially profound implications for the future of tropical forest carbon balance (Phillips et al. 2002, Schnitzer et al. 2021). Lianas are non-self-standing plants that reach the forest canopy by using host tree stems or other established lianas as support, and can compete with trees through local light monopolization, affecting host tree carbon sequestration and growth (Schnitzer and Bongers 2002, Avalos et al. 2007, Paul and Yavitt 2011, van der Heijden et al. 2013, 2015, García León et al. 2018). Lianas have developed root and vascular systems that may effectively compete with trees for water and nutrients (Pérez-Salicrup and Barker 2000, Andrade et al. 2005, Meunier et al. 2021). Furthermore, lianas can cause direct mechanical damage to host trees, causing changes in leaf and branch area index (Schnitzer and Bongers 2002, Reis et al. 2020), factors that could also limit carbon gain. Not surprisingly, liana presence is associated with major decreases in tree productivity, reproduction, biomass accumulation and biomass carbon stocks across tropical forests (Durán and Gianoli 2013, van der Heijden et al. 2015, García León et al. 2017).

In the tropics, liana abundance increases toward areas with lower mean annual precipitation and longer dry seasons. Trees, in contrast, follow the opposite pattern with greater abundance in less seasonal areas (Gentry 1991, DeWalt et al. 2010, Esquivel-Muelbert et al. 2017, Parolari et al. 2020). The growth advantage that lianas have over trees, particularly in areas with high climatic seasonality (Schnitzer and van der Heijden 2019), are suggested to be driven by their more efficient strategy to capture dry-season precipitation (De Deurwaerder et al. 2018), and their efficient hydraulic system, with some studies suggesting lianas also have a safe hydraulic system (Carvalho et al. 2015, Chen et al. 2017, van der Sande et al. 2019). This set of characteristics may allow lianas to absorb and transport more water and potentially enhance their growth rates without risks for their hydraulic system. The enhanced capacity lianas have in keeping water transport efficient, and possibly also safe without restricting growth during the dry season, may be related to the amount and efficiency in use their carbon stores [i.e. non-structural carbohydrates (NSC)] (Schnitzer and van der Heijden 2019). Non-structural carbohydrates are the primary products of photosynthesis, providing plants with the necessary energy for growth and survival (O’Brien et al. 2014, Hartmann and Trumbore 2016). The NSC also play a key role mediating plant responses to abiotic stresses, as they contribute to the regulation of osmotic potential and provide energy for active water transport, thereby contributing to a healthier water balance in plants (Myers and Kitajima 2007, Dietze et al. 2014, Dickman et al. 2015, Thalmann and Santelia 2017). The two main NSC fractions are the soluble sugars (glucose, fructose, sucrose, etc.) that are the main substrate for plant metabolism (e.g. growth, respiration), and starch, a long-term reserve that can be converted to soluble sugars when carbon demand surpasses supply (Rosa et al. 2009, Krasensky and Jonak 2012, MacNeill et al. 2017, Thalmann and Santelia 2017). Although the use of stored NSC has been hypothesized to be involved in the higher capacity lianas have to grow during the dry season compared with trees (Schnitzer and van der Heijden 2019), to our knowledge there is no study that has explored the differences in NSC concentrations in co-occurring trees and lianas (Schnitzer 2018, Schnitzer and van der Heijden 2019).

Liana infestation can hinder tree growth, fecundity and survival (Schnitzer et al. 2005, Ingwell et al. 2010, van der Heijden et al. 2015, García León et al. 2017, McDowell et al. 2018, Reis et al. 2020). Shading caused by lianas over tree canopies can lead to a reduction in the amount of light intercepted by trees, resulting in a decrease in carbon assimilation (Godoy-Veiga et al. 2018). Meanwhile, belowground lianas compete strongly with trees for water and nutrients (Pérez-Salicrup 2001, Andrade et al. 2005, Meunier et al. 2021). The negative response to liana infestation observed in tree growth rates, fecundity and survival, may reflect carbon limitation resulting from decreased carbon gain in host trees caused by above and belowground competition. Such limitation in carbon gain could lead to reduction in NSC reserves which in turn can reduce tree growth and limit its ability to deal with environmental stressors (e.g. drought). Nevertheless, to our knowledge there is no empirical evidence that liana infestation can cause reduction in NSC reserves in long-term storage organs (e.g. stem) of host trees. Given the increase in liana abundance in some tropical areas (Phillips et al. 2002, Schnitzer et al. 2021), it is essential to provide a mechanistic explanation of their impact on tree function, which will enable estimates of future changes in species composition and carbon stocks in tropical forests.

It has been demonstrated that in Amazonian forests the NSC concentrations in tree species can vary along climatic gradients (Signori-Müller et al. 2021), with a higher proportion of soluble sugars in species occurring in drier areas (precipitation <2000 mm year^−1^). The concentration of NSC in Amazonian species can also vary depending on plant life-history strategy (i.e. fast- vs slow-growing species), with slow-growing species presenting higher starch storage and less seasonal variation in NSC concentrations than fast-growing species (Herrera-Ramirez et al. 2021, Signori-Müller et al. 2022). The position of a species along the fast–slow growth continuum may have important implication in plant capacity to deal with stressors, for example Visser et al. (2018) showed that in tropical tree species, liana infestation has stronger negative effects on growth and survival in fast-growing species than in slow-growing species. Fast-growing species, which have lower NSC concentrations than slow-growing species (Herrera-Ramirez et al. 2021, Signori-Müller et al. 2022), may be more vulnerable to liana infestation due to impairment between carbon assimilation and demand for metabolism maintenance. None of these studies, however, have investigated the NSC storage in plants with different life forms (lianas vs trees) and the impact of liana infestation on NSC reserves of host trees. To address this knowledge gap, we sampled trees infested by lianas, the lianas infesting those trees and non-infested trees in two Amazon locations with contrasting mean annual precipitation and dry season length. We quantified starch and soluble sugars, which comprise the most significant portions of NSC reserves in most trees (Martínez-Vilalta et al. 2016). We hypothesized that (i) liana infestations negatively impact the NSC reserves of host trees, with stronger effect in fast-growing species (Visser et al. 2018). Additionally, we expected trees infested by lianas to have lower NSC concentrations than non-infested trees in the dry site, where trees are less adapted to shading (Medina-Vega et al. 2021*b*) and where the forest may already be at their physiological limit (Tavares et al. 2023). (ii) Co-occurring lianas and tress will have different NSC concentrations, with lianas having higher stem NSC concentrations compared with trees in both the dry and moist site, it because lianas have better capacity to intercept light than trees, which can reflect in enhanced carbon gain leading to higher stem NSC concentration on a dry mass basis in wood (Medina-Vega et al. 2021*b*, Medina-Vega et al. 2022).

## Materials and methods

### Site description and species selection

We performed our sampling in two forests in the Amazon with contrasting precipitation regimes. One of the sites is in the Western Amazon, in the Tambopata National Reserve, Puerto Maldonado, Madre de Dios, Peru (12°49′S, 69°16′W), hereafter referred to as the moist site. In this site mean annual precipitation is ≈2450 mm year^−1^, with a 3-month dry season (rainfall <100 mm; Sombroek 2001) extending from June to August (Fick and Hijmans 2017). The other chosen site is an ecotonal forest located at the dry fringe of the Amazon basin, in a permanent plot in Fazenda Vera Cruz, Nova Xavantina, Mato Grosso, Brazil (14°49′S, 52°9′W), hereafter referred as the dry site. In this site mean annual precipitation is ≈1500 mm and the dry season can last up to 6 months (Marimon et al. 2010). We selected these sites due to the marked differences in climatic conditions and consequently species composition and likely functional strategies, in order to identify the difference in NSC storage of co-occurring trees and lianas living in contrasting environments.

Studies with seedlings and adult trees have shown that the stem represents one of the major storage organs for NSC (Poorter and Kitajima 2007, Martínez-Vilalta et al. 2016); therefore, due to financial constraints of sampling and performing chemical analysis we focus our study on the xylem stem of trees and lianas. In both sites, sampling occurred during the wet season, taking place in January 2017 in the moist site and in December 2017 in the dry site. Although stems have smaller diurnal variation in NSC concentrations compared with canopy organs (Tixier et al. 2018), we standardized the sampling time to be between 08:30 and 11:00 a.m. In the field and during the transport to the laboratory, samples were kept on ice. Upon arrival at the laboratory, we microwaved the samples for 90s at 700 W to stop enzymatic processes and oven-dried at ~60 °C for at least 48 h or until they were completely dry.

In each site we sampled trees infested by lianas, the lianas infesting the trees and non-infested trees. We selected the trees based on the liana Crown Occupancy Index (COI, percentage of the canopy covered by lianas, Schnitzer et al. 2005) and tree diameter at breast high (DBH). Here, we considered infested those trees with COI = 3 or 4, meaning that liana cover was ˃50% or 75% of tree canopy cover, respectively. All lianas with DBH ≥5 cm infesting the trees were then sampled too. Non-infested trees are those with COI = 0, meaning that there were no lianas infesting the tree canopy.

In both sites, wherever possible, we sampled individuals with a similar diameter for the trees of the same species, resulting in no difference in diameter between infested and non-infested trees in both sites (Figure S1 available as Supplementary data at *Tree Physiology* Online). In both the moist and dry site we selected late-successional canopy species for NSC sampling. In the moist site all sampled trees had a DBH ≥20 cm. This is a hyperdiverse forest, making it challenging to find many infested and non-infested individuals of the same species. We selected those tree species that are representative of the community and which we could find at least three infested and non-infested individuals (Table 1), the sampled species representing ≈24% of the total plot basal area. In the moist site we sampled 59 individual trees (30 non-infested, 29 infested) from 10 species, and 55 lianas with DBH ≥5 that were infesting those trees. From the 10 species we sampled in the moist site eight are evergreen, one is deciduous (*Pouteria torta*) and one is a semi-deciduous species (*Cedrelinga cateniformis*). For Amazonian species wood density (WD) is a good proxy for life-history strategies (Coelho de Souza et al. 2016). In the moist site, species span a wide range across the fast–slow continuum of growth, with species with WD as low as 0.38 g cm^−3^ to up to 0.87 g cm^−3^ (Chave et al. 2009, Zanne et al. 2009).

**Table 1:** Collected species and number of infested and non-infested trees, in the dry and in the moist site.

Species	Site	*n*	WD^1^	Phenology
*Amaioua guianensis Aubl.*	Dry	Infested = 3	0.67	Evergreen
		Non-infested = 5		
*Brosimum rubescens Taub.*	Dry	Infested = 11	0.80	Evergreen
		Non-infested = 9		
*Chaetocarpus echinocarpus (Baill.) Ducke*	Dry	Infested = 11	0.79	Evergreen
		Non-infested = 4		
*Ephedranthus parviflorus S.Moore*	Dry	Infested = 6	0.72	Evergreen
		Non-infested = 6		
*Mabea fistulifera Mart.*	Dry	Infested = 9	0.61	Brevi-deciduous
		Non-infested = 7		
*Calophyllum brasiliense Cambess.*	Moist	Infested = 3	0.58	Evergreen
		Non-infested = 3		
*Cedrelinga cateniformis (Ducke) Ducke*	Moist	Infested = 3	0.50	Semi-deciduous
		Non-infested = 3		
*Eschweilera coriacea (DC.) S.A.Mori*	Moist	Infested = 3	0.85	Evergreen
		Non-infested = 3		
*Hymenaea parvifolia Huber*	Moist	Infested = 3	0.87	Evergreen
		Non-infested = 3		
*Hymenopus heteromorphus (Benth.) Sothers & Prance* ^2^	Moist	Infested = 3	0.81	Evergreen
		Non-infested = 3		
*Micropholis guyanensis (A.DC.) Pierre*	Moist	Infested = 2	0.65	Evergreen
		Non-infested = 3		
*Pourouma guianensis Aubl.*	Moist	Infested = 3	0.38	Evergreen
		Non-infested = 3		
*Pourouma minor Benoist*	Moist	Infested = 3	0.43	Evergreen
		Non-infested = 3		
*Pouteria torta (Mart.) Radlk.*	Moist	Infested = 3	0.76	Deciduous
		Non-infested = 3		
*Protium altissimum (Aubl.) Marchand* ^3^	Moist	Infested = 3	0.70	Evergreen
		Non-infested = 3		

^1^WD: wood density (g cm^−3^).

^2^Former *Licania heteromorpha Benth..*

^3^Former *Tetragastris altissima (Aubl.) Swart.*

In the dry site, due to the smaller tree sizes, sampling was performed on trees with diameter ≥10 cm. However, preference was given to larger individuals whenever possible. In the dry site, we sampled individuals from four species from a mixed plot where these species accounted for ≈61% of the total plot basal area (Soares Jancoski et al. 2022), and one species, *Brosimum rubescens*, from a plot where it is the monodominant species and accounts for ≈70% of the total plot basal area. For the dry site we sampled in total 71 trees (31 non-infested trees, 40 infested trees) and 37 lianas with DBH ≥5 cm infesting those trees. In the dry site four out five species are evergreen, and one is brevi-deciduous (*Mabea fistulifera*). Species sampled in this site have high WD, varying between 0.61 and 0.80 g cm^−3^ (Soares Jancoski et al. 2022). Due to the difficulty in accessing leaf and fertile material, lianas were not identified on any of the sites.

We collected stem samples for both trees and lianas at 1.20 m above the ground using a 4.3 mm increment borer (Haglöf Company Group, Sweden). To quantify the stored NSC concentration and not transient sugars we removed the bark and phloem and then obtained the stem xylem cores of lianas and trees. As tree species vary in their growth rates, we use a proportion of the sampled core that should roughly represent the last 5 years of growth increment. This is because liana infestation status (COI) changes over time and we established 5 years as the minimum period over which COI measured during our field campaign could reasonably be expected to apply. Establishing a fixed window for analysis based on 5 years of growth should also reduce the bias caused by trees growing under different environmental conditions. To estimate the proportion of the core to use in the NSC analyses we calculated the growth rate of each species using inventory data from TAM-05, TAM-07, VCR-01 and VCR-02 plots (Lopez-Gonzalez et al. 2011, ForestPlot.net et al. 2021). When possible, we used the growth rate of the sampled individual trees to estimate the amount of material for NSC analysis. When this was not possible, we used the mean growth rate calculated for the species across all individuals of the species occurring in the plot. It is worth noting that the sampled infested and non-infested trees did not differ in growth rate, likely due to a temporal mismatch between the growth data at our disposal and the liana infestation data (data not presented). This result may reflect the small number of individuals of each species, and the fact that for some of them it was not possible to obtain the mean growth rate. Lianas have a small stem diameter increment (Putz 1990, Restom and Nepstad 2004), and therefore, we standardized the amount of material used to 1.5cm long cores (excluding bark and phloem) to ensure that we had enough liana material for the NSC analysis.

### Non-structural carbohydrates quantification

Before the NSC quantification we ground the samples to a fine powder (GenoGrinder®, USA). Non-structural carbohydrates are defined here as free, low molecular weight sugars (glucose, fructose, sucrose, etc.) plus starch. Non-structural carbohydrates were analyzed as described in Hoch et al. (2002) with minor modifications (Rowland et al. 2015, Signori-Müller et al. 2021, 2022). First, we diluted 15 mg of the ground plant material with 1.6 mL of distilled water and then incubated in a water bath at 90–100 °C for 60 min to solubilize sugars. We then took an aliquot of 700 μL from each sample and used the remaining aliquot volume (900 μL) to determine soluble sugar concentrations using invertase from *Saccharomyces cerevisiae* (Sigma-Aldrich, USA) to break down sucrose and fructose to glucose. Additionally, for both reaction routines, we used GAHK (Glucose Assay Hexokinase Kit—Sigma-Aldrich, USA) together with phosphoglucose isomerase from *S. cerevisiae* (Sigma-Aldrich, USA). The concentration of free glucose was measured photometrically in a 96-well microplate spectrophotometer at 340 mm (EPOCH—Biotek Instruments INC, USA). The 700 μL aliquot that we initially separated was incubated overnight to react with amyloglucosidase from *Aspergillus niger* (Sigma-Aldrich, USA) to break down the total NSC to glucose. Thereafter, total glucose (corresponding to NSC) was determined as described above and starch was calculated as total NSC minus soluble sugars. All NSC values are expressed in mg g^−1^ dry mass.

### Statistical analysis

We performed all statistical analysis using R software (R Core Team 2018, version 4.2.3). Preliminary tests included: analysis of normality (Shapiro–Wilk) and homogeneity of variances (Flinger–Killeen) for each NSC fraction (NSC, starch and soluble sugars). As NSC, starch and soluble sugars were not normally distributed, we used non-parametric analyses or log1p transformed the data.

To conduct a paired analysis to test differences in median stem NSC, starch and soluble sugars between infested and non-infested trees we averaged the concentration per species and used the Paired Samples Wilcoxon Test. We also tested the effect of infestation at species level for species where *n* ≥ 3 (Table 1) using a *t*-test, following log1p transformation of NSC data. We investigated whether infestation influences NSC concentration of trees depending on the leaf habit. Due to the small number of species that are not evergreen (Table 1), we used the individual value of each sampled tree and grouped deciduous and semi-deciduous species into a single group. Analysis was conducted independently for each site and NSC fraction using Wilcoxon Tests.

As species with different life strategies may differ in their response to liana infestation (Visser et al. 2018), we tested how the NSC varied depending on the level of infestation (COI) interacting with species WD, a trait established as a good proxy to identify the position of species across the fast–slow continuum of growth for Amazonian species (Kitajima and Poorter 2008, Coelho de Souza et al. 2016). We performed the analysis separately for each site and each NSC fraction using the *lmer* function from lme4 package (Bates et al. 2015); for this analysis we used the individual log1p transformed NSC, starch and soluble sugars concentration for each tree, and species as random effect. Wood density data for the dry site are from Soares Jancoski et al. (2022) and for the moist site from Zanne et al. (2009).

To compare the NSC concentration of trees and lianas we used Wilcoxon rank sum test. Analysis was performed using the individual NSC concentration of each individual tree and liana, for each NSC fraction and site. As lianas were not identified it was not possible to carry out analysis that control for the lack of independence between observations within species. For all analyses, we assumed a significance level of 0.05.

## Results

### Infested versus non-infested trees

**Figure 1:**
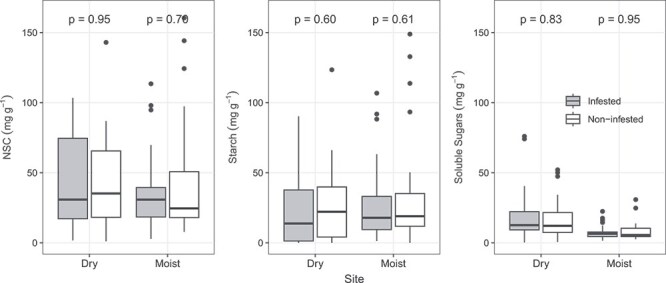
Concentrations of stem NSC, starch and soluble sugars in co-occurring trees species with liana infestation >50% (grey) and in trees without liana infestation (white). For figure and analysis, we used the mean concentration per species. Differences between groups were tested using paired samples Wilcoxon test. Data are present for the dry and moist site. Dry site: infested *n* = 40, non-infested *n* = 31; moist site: infested *n* = 29, non-infested *n* = 30. Each box encompasses the 25th to 75th percentiles; the median is indicated by the horizontal line within each box while external vertical lines indicate the 10th and 90th percentiles; dots indicate outliers.

**Figure 2:**
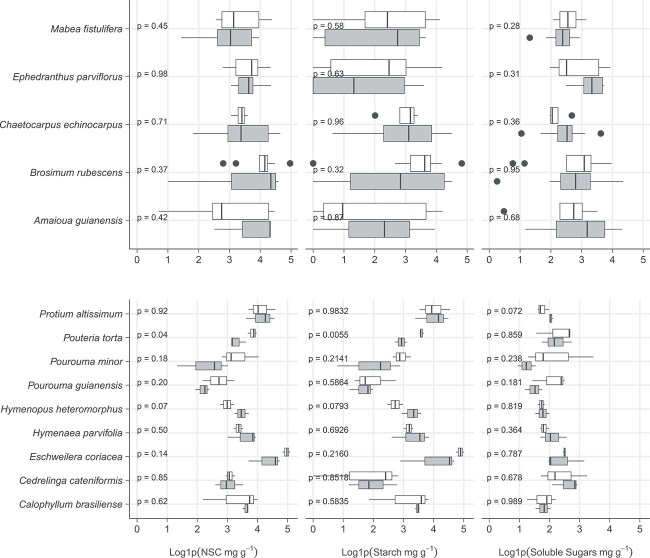
Concentrations of stem NSC, starch and soluble sugars from trees infested by lianas (grey) and non-infested trees (white). Data are presented for the dry site (top panels) and moist site (bottom panels). *Micropholis guyanensis* was excluded due to small sample size (*n* < 3). Data were log1p transformed and differences between groups were tested using *t*-test. Each box encompasses the 25th to 75th percentiles; the median is indicated by the vertical line within each box while external horizontal lines indicate the 10th and 90th percentiles.

Our results demonstrate that NSC concentrations in stem xylem are similar in infested (Median ± SE; 30.8 ± 1.73 mg g^−1^) and non-infested trees (28.4 ± 1.43 mg g^−1^; *P* > 0.05; Paired Samples Wilcoxon Test; Figure S2 available as Supplementary data at *Tree Physiology* Online). Furthermore, these results remain consistent across both the dry and moist sites (Figure 1). Infested and non-infested trees have similar starch (infested: 16.0 ± 1.92 mg g^−1^; non-infested: 1.32 ± 1.31 mg g^−1^) and soluble sugars (infested: 7.67 ± 1.35 mg g^−1^; non-infested: 7.88 ± 0.75 mg g^−1^) concentrations (Figure S2 available as Supplementary data at *Tree Physiology* Online), with results consistent for the dry and moist sites (Figure 1). At species level (Figure 2), only *P. torta*, from the moist site, showed statistically significant differences in stem starch concentration between infested (17.8 ± 2.17 mg g^−1^) and non-infested trees (34.7 ± 1.55, *P* < 0.05); all other species from both sites had similar stem NSC, starch and soluble sugars concentrations between infested and non-infested trees. There were also no significant differences observed between infested and non-infested trees when we examined deciduous and evergreen species separately (Figure S3 available as Supplementary data at *Tree Physiology* Online).

**Figure 3:**
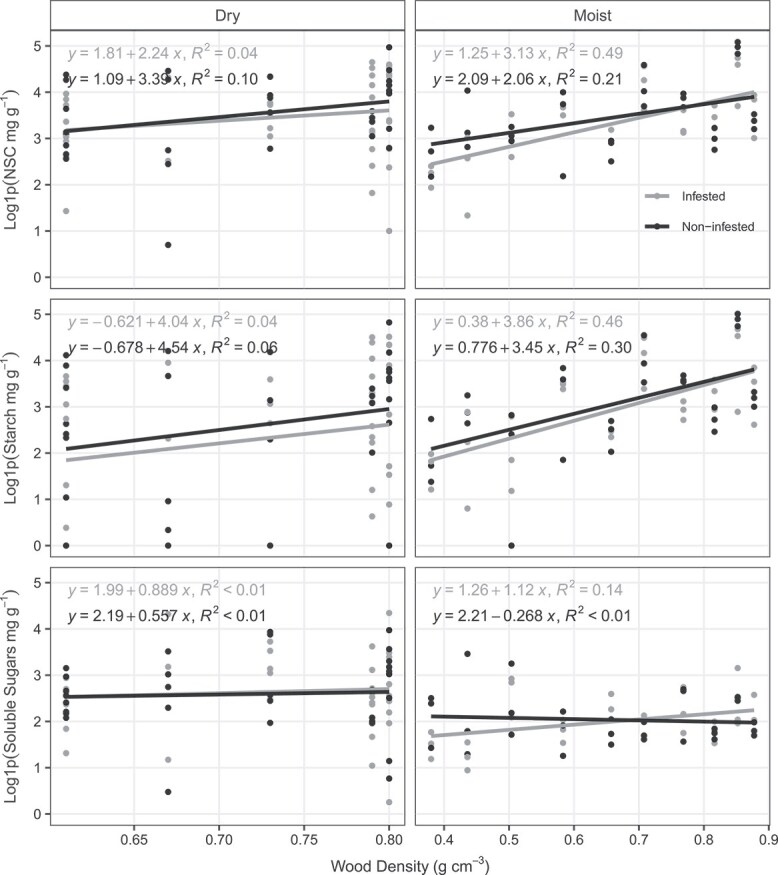
Relationship between stem NSC, starch and soluble sugars (mg g^−1^) with WD (g cm^−3^). Mean WD per species in the dry site (*n* = 5 species) are from Soares Jancoski et al. (2022) and for the species in moist site (*n* = 10 species) we used the basin mean from Zanne et al. (2009). The R^2^ values were calculated using ordinary linear regression, NSC concentration and its fraction were log1p transformed before analysis. Gray color represents infested trees and black color non-infested trees.

We fitted Linear Mixed-Effects Models to predict NSC, starch and soluble sugar concentration with infestation level and WD as predictors and found that overall, the explanatory power of these models was low, especially at the dry site (Tables 2 and 3). Interaction between infestation level (COI) and WD did not explain the variance in any NSC fraction in both dry and moist sites (Figure S4 available as Supplementary data at *Tree Physiology* Online). Only WD alone in the moist site has a statistically significant and positive relationship on stem NSC and starch concentration (Figure 3; Tables 2 and 3).

**Table 2:** Linear mixed-effects models results to predicting NSC, starch and soluble sugars with crown occupancy index (COI) and wood density (WD) in the dry site.

	log1p (NSC)			log1p (starch)			log1p (soluble sugars)		
Predictors	Estimates	CI	*P*	Estimates	CI	*P*	Estimates	CI	*P*
(Intercept)	1.46	−1.44 to 4.35	0.319	−0.62	−5.83 to 4.58	0.812	2.44	−0.90 to 5.77	0.149
COI	0.03	−1.04 to 1.11	0.952	0.03	−1.87 to 1.94	0.972	−0.25	−1.28 to 0.78	0.633
WD	2.85	−1.16 to 6.86	0.161	4.39	−2.82 to 11.60	0.228	0.19	−4.43 to 4.82	0.933
COI × WD	−0.07	−1.56 to 1.41	0.923	−0.13	−2.76 to 2.50	0.922	0.37	−1.05 to 1.79	0.603
Random effects									
σ^2^	0.77			2.42			0.70		
τ_00_	0.00_Species_			0.01_Species_			0.05_Species_		
ICC				0.00			0.07		
N	5_Species_			5_Species_			5_Species_		
Observations	71			71			71		
Marginal R^2^/conditional R^2^	0.055/NA^1^			0.043/0.047			0.013/0.080		

^1^Not estimated because variance estimates for random effect is nearly zero.

**Table 3:** Linear mixed-effects models results to predicting NSC, starch and soluble sugars with crown occupancy index (COI) and wood density (WD) in the wet site. Bold values represent significative relationships.

	log1p (NSC)			log1p (starch)			log1p (soluble sugars)		
Predictors	Estimates	CI	P	Estimates	CI	P	Estimates	CI	P
(Intercept)	2.06	0.72 to 3.39	**0.003**	0.73	−0.98 to 2.43	0.396	2.18	1.27 to 3.10	**<0.001**
COI	−0.24	−0.55 to 0.06	0.110	−0.10	−0.51 to 0.31	0.629	−0.27	−0.58 to 0.03	0.075
WD	2.14	0.17 to 4.10	**0.033**	3.55	1.04 to 6.05	**0.006**	−0.19	−1.53 to 1.16	0.781
COI × WD	0.29	−0.15 to 0.72	0.194	0.08	−0.52 to 0.68	0.795	0.37	−0.06 to 0.81	0.093
Random effects									
σ^2^	0.23			0.43			0.23		
τ_00_	0.19_Species_			0.30_Species_			0.05_Species_		
ICC	0.46			0.41			0.18		
N	10_Species_			10_Species_			10_Species_		
Observations	59			59			59		
Marginal R^2^/conditional R^2^	0.332/0.637			0.352/0.616			0.062/0.230		

### Trees versus lianas

Due to the lack of differences in NSC, starch and soluble sugar concentrations between infested and non-infested trees we grouped them to compare with lianas.

**Figure 4:**
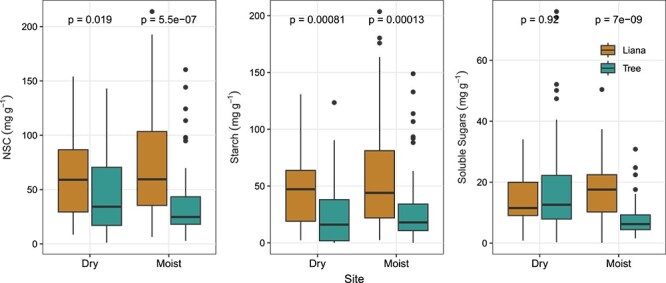
Concentrations of stem NSC, starch and soluble sugars in co-occurring lianas (brown) and trees (green). Data are present for the dry and moist site. To test for differences between life form within site we used Wilcoxon rank sum test. Dry site: liana *n* = 37, tree *n* = 71; moist site: liana *n* = 73, tree = 59 individuals. Each box encompasses the 25th to 75th percentiles; the median is indicated by the horizontal line with in each box while external vertical lines indicate the 10th and 90th percentiles; dots indicate outliers.

Lianas have higher stem NSC concentrations than trees (Figure 4) both in the dry (liana: 59.1 ± 6.15 mg g^−1^; tree: 34.2 ± 3.69 mg g^−1^; *P* < 0.001; Wilcoxon rank sum test) and moist site (liana: 59.5 ± 6.15 mg g^−1^; tree: 24.8 ± 4.43 mg g^−1^; *P* < 0.001). Stem starch concentrations were higher in lianas than in trees (Figure 4), both in the dry (liana: 47.2 ± 5.56 mg g^−1^; tree: 16 ± 3.30 mg g^−1^; *P* < 0.001 ) and moist site (liana: 44 ± 6.69 mg g^−1^; tree: 17.9 ± 4.33 mg g^−1^; *P* < 0.001). In the moist site, stem soluble sugar concentrations were higher in lianas (17.6 ± 1.31 mg g^−1^; *P* < 0.001) than in trees (6.25 ± 0.73 mg g^−1^; Figure 4), while in the dry site both life forms have similar stem soluble sugar concentrations (liana: 11.5 ± 1.24 mg g^−1^; tree: 12.6 ± 1.83 mg g^−1^; *P* = 0.92). Lianas had similar NSC, starch and soluble sugar concentrations in both sites (Figure S5 available as Supplementary data at *Tree Physiology* Online), while trees only differed among sites with respect to soluble sugar concentrations, which was higher in the dry site (Figure S5 available as Supplementary data at *Tree Physiology* Online).

**Figure 5:**
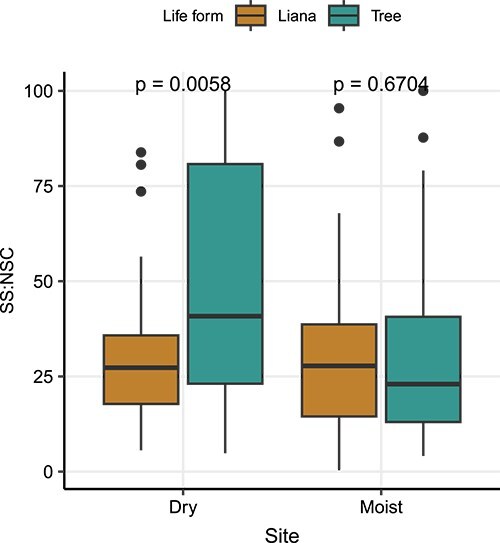
Comparison of proportion of NSC in the form of soluble sugars (SS:NSC) in stems of co-occurring lianas (brown) and trees (green). Data are present for the dry and moist site. To test for differences between life-form within site we used Wilcoxon rank sum test. Dry site: liana *n* = 37, tree *n* = 71; moist site: liana *n* = 73, tree = 59 individuals. Each box encompasses the 25th to 75th percentiles; the median is indicated by the horizontal line with in each box while external verticallines indicate the 10th and 90th percentiles; dots indicate outliers.

The proportion of total NSC allocated to soluble sugars (SS:NSC) was similar between lianas (30.0 ± 2.67) and trees (29.3 ± 2.75) in the moist site (*P* = 0.60; Wilcoxon rank sum test), but not in the dry site (Figure 5), where we find trees have higher SS:NSC (49.1 ± 3.73) than lianas (30.9 ± 3.16; *P* = 0.005). Intersite comparisons show that lianas have similar SS:NSC among sites (*P* > 0.05), while for trees the higher SS:NSC are found in the dry site (*P* < 0.001; Figure S6 available as Supplementary data at *Tree Physiology* Online).

## Discussion

Despite its role in plant fitness, to our knowledge, this is the first study to investigate differences in NSC concentrations of trees and lianas, and the effect of liana infestation on trees' NSC concentrations. Even though liana infestation can have negative impact on host trees (Schnitzer et al. 2005, 2014; van der Heijden et al. 2013, 2015; Reis et al. 2020), our results do not support the hypothesis that liana infestation can lead to changes in stem NSC concentration of Amazonian trees (Figure 1).The hypothesis that lianas have higher stem xylem NSC than trees was confirmed by our results (Figure 2). However, our hypothesis that major differences in NSC concentration between trees and lianas would occur in the dry site was not confirmed by our results. In fact, in the dry site, soluble sugar concentrations in tree stem xylem were indistinguishable from those from lianas, while starch and NSC were higher in lianas than in trees in both sites (Figure 4).

### Infested versus non-infested trees

Liana presence may prevent trees from experiencing the expected maximum growth capacity throughout their lifetime (Godoy-Veiga et al. 2018), with more pronounced negative effect in fast-growing species than in slow-growing species (Visser et al. 2018). Despite that we found no difference in stem NSC, starch and soluble sugar concentration of infested and non-infested trees regardless of tree life-history strategy (Tables 1 and 2, Figure 3, Figure S4 available as Supplementary data at *Tree Physiology* Online). The fitted linear models only showed a significant relationship between NSC and starch with WD in the moist site (Figure 3), and it was independent of infestation level. These results are in line with previous studies with Amazonian species, which showed that across the fast–slow continuum of life-history strategies, stem starch is positively related with WD (Herrera-Ramirez et al. 2021, Signori-Müller et al. 2022). The lack of relationship between starch and WD in the dry site may reflect the fact that only species with high WD were sampled there. Reis et al. (2020) showed that in the southern Amazon, where our dry site is located, slow-growing, dense-wooded species are more susceptible to liana infestation than fast-growing species, elucidating the absence of species with low WD in our data set. Considering the many impacts lianas have on trees’ performance and community structure, our results are surprising (Ingwell et al. 2010, García León et al. 2017, Reis et al. 2020).

Although lianas can exert mechanical damage on host trees, causing changes in leaf and branch area index (Schnitzer and Bongers 2002, Reis et al. 2020), the mechanical stress they induce on host trees does not affect stem NSC concentrations (Figure 1). Rademacher et al. (2021) manipulated phloem transport in a conifer species through compressing the stem, and found that although compression affects wood formation it does not affect the NSC reserves. Investigating the impact of liana infestation on water status of trees species in a forest close to our dry site, in southeast Amazon, Beú (2019) found infested and non-infested trees have similar pre-dawn and mid-day water potential across seasons. Based on our findings of no difference in NSC concentrations between infested and non-infested trees, we hypothesize that the much-reported impacts of lianas on forest dynamics (reduced growth, increased mortality) (Ingwell et al. 2010, van der Heijden et al. 2015, Reis et al. 2020, 2022) may be mediated primarily by the weakening of tree structure.

### Trees versus lianas

Trees can have access to NSC pools integrating carbon accumulated over more than a decade (Vargas et al. 2009, Muhr et al. 2018). Despite the ability of trees to use old, stored carbon, some NSC accumulated in woody tissues may become sequestered, and therefore not available for future use, representing a metabolic dead end, with the carbon no longer physiologically active and so not affecting metabolism (Millard et al. 2007). Maximum ages of accessible carbon reserves could be affected by the time that sapwood is alive and functional before it undergoes heartwood transformation (Muhr et al. 2018). Average sapwood lifespans for tropical tree species can vary between 5.7 and 88.6 years with an average of 29.78 years (van der Sande et al. 2015). In the trees, we quantified the NSC concentration in a portion of the xylem that represented the increment of the last 5 years (see Materials and methods), which likely encompasses a functional portion of the xylem. For lianas, we standardized the length of the xylem segment we used for analysis to 1.5 cm, which may represent growth increments of > 10 years, considering stem increment of 1.4 mm year^−1^ presented by Putz (1990) and Restom and Nepstad (2004). Information about liana sapwood lifespan is missing, however it is known that they have a high sapwood to heartwood ratio (Tyree and Ewers 1996), therefore for the purposes of this study we can assume that the xylem portion we analyzed for lianas is active and NSC reserves in this portion accessible to be used. To understand the liana dynamics and its increase in abundance in some areas, we must comprehend its carbon metabolism, including the dynamics of NSC, which remains unexplored compared with the water relations (i.e. hydraulic traits). Future studies should prioritize investigating the lifespan of liana xylem and the extent to which lianas can utilize carbon stores (NSC) that are several years old, and on which temporal scale it occurs (seasonally vs extreme events) (Carbone et al. 2007, Vargas et al. 2009).

Parenchymatic cells constitute a major NSC storage compartment in plants (Plavcová and Jansen 2015), and have been reported to be found in lianas in amounts about twice as high as in angiosperm trees (Morris et al. 2016). We find the stem xylem NSC concentrations in lianas are double those in trees. This may be driven by a greater parenchyma fraction in stem xylem of lianas relative to trees and not necessarily by greater NSC concentrations per unit of parenchyma.

A recent study conducted in a tropical forest in Panama showed that lianas have the ability to maintain higher growth rates during dry season than trees, the authors speculate this may occur through the maintenance of high water potential or by relying on stored NSC (Schnitzer and van der Heijden 2019). Starch concentrations, which we found to be higher in lianas than in trees in both sites, is a long-term NSC fraction that can be remobilized to fulfill plant needs for carbon when demand is higher than assimilation (MacNeill et al. 2017). Through analysis of scanning electron photomicrographs of liana xylem, Masrahi (2014) found a dense accumulation of starch grains on ray parenchyma cells near vessel groups of lianas from a very dry area (precipitation 150 mm year^−1^). We speculate that by relying on the structural investment of trees for mechanical support, lianas can allocate a high proportion of the assimilated carbon into reserves. By being hydrolyzed into soluble sugars, starch can support growth, enhance water flow by raising vessel osmotic pressure to regulate conductance and reduce the risk of embolism entering the vessels (Thalmann and Santelia 2017, Tomasella et al. 2017). High concentrations of starch allied to parenchyma cells in contact with vessels could enable lianas to have easy access and use of the NSC reserves, increasing hydraulic efficiency without compromising safety (Secchi et al. 2017), promoting growth during period of carbon limitation and fuelling liana reproductive events and leaf flush (Olson 2003, Schnitzer and van der Heijden 2019).

In this study differences between trees and lianas were overall consistent in both dry and moist site. Other studies however, found that co-occurring trees and lianas differ more in drier than in wetter sites in relation to their functional traits involved in water transport, (Medina-Vega et al. 2021*a*, Smith-Martin et al. 2022). The only similarity observed in stem NSC concentration between the different life forms was found in the soluble sugars fraction in the dry forest (Figure 4). Although absolute stem soluble sugar concentration of both life-forms was similar in the dry site, the proportion of the total NSC allocated to soluble sugars (SS:NSC) was higher for trees than for lianas (Figure 5). A study considering leaves and branches of Amazonian tree species also found higher SS:NSC in more dry and seasonal sites (Signori-Müller et al. 2021). Together these results reinforce the idea that soluble sugars cannot be drawn below a certain threshold (Sala et al. 2012) due to their immediate role in the maintenance of plant metabolism (e.g. growth, respiration, osmoregulation, embolism repair, etc; Rosa et al. 2009, Krasensky and Jonak 2012, MacNeill et al. 2017, Thalmann and Santelia 2017). Based on the higher starch concentrations in lianas than in trees, lianas could have more carbon to fulfill their soluble sugar requirements than trees, in both dry and moist sites. The increase in liana abundance toward areas that are experiencing reduction in water availability and increasing seasonality, especially in the Neotropics (Phillips et al. 2002, Laurance et al. 2014, Marimon et al. 2020), may be the result of a well-adjusted hydraulic system and carbon metabolism (McDowell 2011).

## Conclusions

We do not rule out the hypothesis that liana infestation may impact the NSC dynamics of host trees. To better understand whether this is the case, future studies should investigate for example whether there are differences in NSC concentrations of infested and non-infested trees also during the dry season, when water transport and carbon assimilation are potentially compromised (Wagner et al. 2016). Leaves and branches, which have more dynamic NSC pools, should also be considered (Würth et al. 2005, Signori-Müller et al. 2022). It may be that the canopy organs have more carbon imbalance due to liana infestation than the stem xylem, which can serve as a long-term storage organ and thus less affected if storage is prioritized over growth (Herrera-Ramirez et al. 2021, Signori-Müller et al. 2022).

In Amazonian tree species, high starch concentration in stem is linked to high WD and low mortality rates (Herrera-Ramirez et al. 2021, Signori-Müller et al. 2022). For lianas, it is unknown whether starch or soluble sugar concentrations are related to plant life-history traits and whether the seasonal dynamics are similar to those in trees. An increasing number of experimental studies with mature trees, seedlings and shrubs shows that under stressful conditions plants with higher NSC concentrations can cope better with stressors (e.g. drought, canopy damage), hence increasing survival rates (e.g. O’Brien et al. 2014, Shibata et al. 2016, Tomasella et al. 2017, Gessler and Grossiord 2019, Guo et al. 2020). Non-structural carbohydrates may play a similar role in liana response to stressors as they do in plants with other life forms, even favoring liana growth in periods when trees would prioritize the maintenance of storage (Chuste et al. 2019, Schnitzer and van der Heijden 2019). The high starch concentration in lianas compared with trees points to differences in carbon gain (Cai et al. 2009) and possibly in carbon storage and use. To be able to predict changes in forest composition and carbon accumulation, we need to understand the mechanisms linked to lianas’ ability to increase in abundance in areas where seasonality in precipitation is increasing. An underrated but key component to understand it could be related to how lianas use their carbon reserves and how well coordinated it is with their hydraulic system. Despite their significance, lianas are persistently understudied and even a basic understanding of NSC dynamics remains elusive (Zotz et al. 2006, Slot et al. 2014).

## Supplementary Material

Supplementary_information_SignoriMuller_2023_TreePhys_tpad096

## Data Availability

The stem non-structural carbohydrate concentration data for trees and lianas are available as data packages via ForestPlots.net (Signori-Müller et al. 2023). The inventory data to estimate species growth rate at the study site are from the RAINFOR network, available upon request at ForestPlots.net (Lopez-Gonzalez et al. 2011, ForestPlot.net et al. 2021). Wood density from species occurring in the dry site are from Soares Jancoski et al. (2022), for species in the moist site, we used mean species WD for the Amazon Basin from Chave et al. (2009) and Zanne et al. (2009).
