# Supplementary material for: Tropical forest lianas have greater non-structural carbohydrate concentrations in the stem xylem than trees
Source: Tree Physiol. 2023 Aug 16;44(13):159–72. doi: 10.1093/treephys/tpad096 (PMC12086676; doi:10.1093/treephys/tpad096)
Supplement: Supplementary_information_SignoriMuller_2023_TreePhys_tpad096 [file supplementary_information_signorimuller_2023_treephys_tpad096.pdf]

Supplementary information for

Tropical forest lianas have greater non-structural carbohydrate concentrations in the stem xylem than trees

Caroline Signori-Müller*, David Galbraith, Julia Valentim Tavares, Simone Matias Reis, Francisco Carvalho Diniz, Martin Gilpin, Beatriz Schwantes Marimon, Geertje M. F. van der Heijden, Camila Borges , Bruno Barçante Ladvocat Cintra, Sarah Mião, Paulo S. Morandi, Alex Nina, Carlos A. Salas Yupayccana, Manuel J. Marca Zevallos, Eric G. Cosio, Ben Hur Marimon Junior, Abel Monteagudo Mendoza, Oliver Phillips, Norma Salinas, Rodolfo Vasquez, Maurizio Mencuccini[1] and Rafael S. Oliveira

* Correspondence author: c.signori-muller@exeter.ac.uk

https://doi.org/10.1093/treephys/tpad096

Resumo (Português)

Lianas são importantes componentes das florestas tropicais, conhecidas por competir com as árvores hospedeiras por recursos, diminuindo o crescimento aumentar a mortalidade das árvores. Diante do aumento na abundância de lianas em algumas florestas e de seus impactos na função florestal, uma compreensão integrada da dinâmica de carbono das lianas e das árvores infestadas por lianas é fundamental para uma melhor previsão das respostas das florestas tropicais às mudanças climáticas. Os carboidratos não estruturais (NSC) são o principal substrato para o metabolismo das plantas (ex., crescimento, respiração), e têm sido associados à capacidade das árvores de sobreviver em condições de estresse ambiental, porém pouco se sabe sobre como os NSC variam entre diferentes formas de vida ou sobre como a infestação por lianas afeta os NSC das árvores hospedeiras. Quantificamos as concentrações de NSC total (NSC) e suas frações (amido e açúcares solúveis) no xilema do tronco de árvores sem infestação por lianas, árvores com mais de 50% da copa coberta por lianas e nas lianas infestando essas árvores. Nossa hipótese foi de que i) a infestação por lianas resulta em uma redução no armazenamento de NSC nas árvores hospedeiras devido à diminuição na assimilação de carbono causada pela competição por recursos (ex. água, nutrientes, luz); ii) árvores e lianas, que diferem muito em características funcionais relacionadas ao transporte de água e absorção de carbono, também teriam grandes diferenças no armazenamento de NSC. Como a disponibilidade de água desempenha um papel significativo na dinâmica de NSC das espécies arbóreas amazônicas, testamos essas hipóteses em um sítio úmido na Amazônia Ocidental e em um sítio mais seco na Amazônia Meridional. Não encontramos diferenças nas concentrações de NSC, amido ou açúcares solúveis entre árvores infestadas e não infestadas, em nenhum dos sítios. Esse resultado sugere que o impacto negativo das lianas nas árvores pode ser mediado por mecanismos diferentes da depleção das concentrações de NSC das árvores hospedeiras. Os nossos resultados mostram que o amido, uma reserva de NSC de longo prazo, apresentou diferenças entre as formas de vida nos dois sítios investigados, o que reflete as variações no ganho e no uso de carbono entre lianas e árvores. As concentrações de açúcares solúveis foram mais altas nas lianas do que nas árvores no sítio úmido, mas não diferiram entre as formas de vida no sítio seco. A falta de diferença nos açúcares solúveis entre árvores e lianas no sítio seco enfatiza a importância dessa fração de NSC para o metabolismo das plantas que ocorrem em ambientes limitados em água.

Palavras-chave: amido, açúcares solúveis, infestação por lianas, árvore hospedeira

Resumen (Español)

Las lianas son importantes componentes de los bosques tropicales, conocidas por competir con los árboles hospederos por recursos, reduciendo su crecimiento y aumentando la mortalidad de los árboles. Ante el aumento observado en la abundancia de lianas en algunos bosques y sus impactos en la función forestal, es fundamental comprender de manera integrada la dinámica del carbono de las lianas y los árboles infestados por lianas para predecir de manera más precisa las respuestas de los bosques tropicales al cambio climático. Los carbohidratos no estructurales (NSC) son el principal sustrato para el metabolismo de las plantas (por ejemplo, crecimiento, respiración) y se ha observado que están relacionados con la capacidad de los árboles de sobrevivir en condiciones de estrés ambiental. Sin embargo, se sabe poco acerca de cómo varían los NSC entre diferentes formas de vida y cómo la infestación por lianas afecta los NSC de los árboles hospederos. Cuantificamos las concentraciones de NSC total (NSC) y sus fracciones (almidón y azúcares solubles) en la xilema del tronco en árboles sin infestación de lianas, árboles con más del 50% de la copa cubierta por lianas y las lianas que infestan esos árboles. Nuestra hipótesis fue que i) la infestación por lianas conllevar una reducción en el almacenamiento de NSC en los árboles hospederos debido a la disminución en la asimilación de carbono causada por la competencia por recursos (agua, nutrientes, luz); ii) los árboles y las lianas, que difieren significativamente en características funcionales relacionadas con el transporte de agua y la absorción de carbono, también tendrían grandes diferencias en el almacenamiento de NSC. Dado que la disponibilidad de agua desempeña un papel importante en la dinámica de los NSC de las especies arbóreas amazónicas, pusimos a prueba estas hipótesis en un sitio húmedo en la Amazonía Occidental y en un sitio más seco en la Amazonía Meridional. No encontramos diferencias en las concentraciones de NSC, almidón o azúcares solubles entre los árboles infestados y no infestados por las lianas, en ninguno de los sitios. Este resultado sugiere que el impacto negativo de las lianas en los árboles puede ser mediado por mecanismos diferentes a la reducción de las concentraciones de NSC en los árboles hospederos. Nuestros resultados muestran que el almidón, que es una reserva de NSC de largo plazo, presentó diferencias entre las formas de vida en ambos sitios investigados, lo que refleja las variaciones en la captación y utilización de carbono entre las lianas y los árboles. Las concentraciones de azúcares solubles fueron más altas en las lianas que en los árboles en el sitio húmedo, pero no mostraron diferencias entre las formas de vida en el sitio seco. La falta de diferencia en los azúcares solubles entre los árboles y las lianas en el sitio seco resalta la importancia de esta fracción de NSC para el metabolismo de las plantas que se encuentran en ambientes limitados en agua.

Palabras clave: almidón, azúcares solubles, infestación de lianas, árbol hospedero

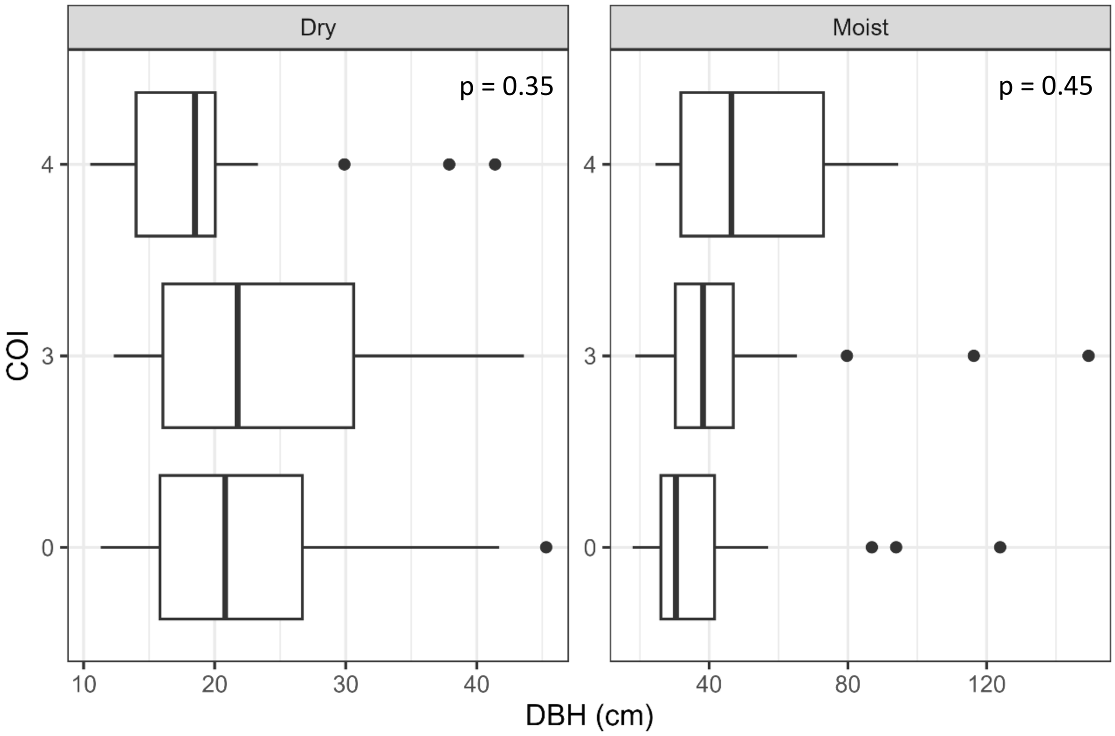

Figure S1 – Crown occupancy index (COI) plotted against tree diameter at breast height (DBH) for the dry and moist site. COI = 0 represent those trees without liana infestation, COI = 3 or 4 meaning that liana cover was more than 50% or 75% of tree canopy cover, respectively. To test for differences in diameter among levels of infestation (COI) we used Kruskal-Wallis rank sum test. Each box encompasses the 25th to 75th percentiles; the median is indicated by the horizontal line within each box while external horizontal lines indicate the 10th and 90th percentiles; dots indicate outliers.

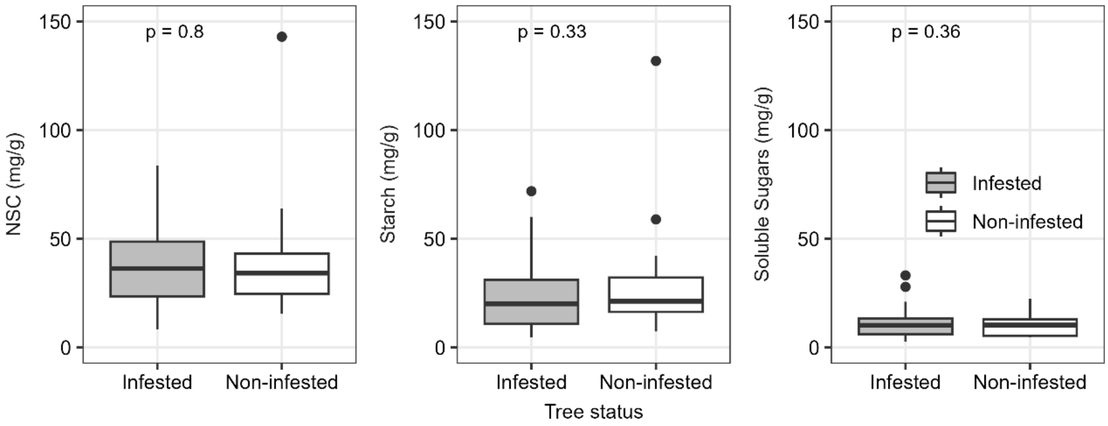

Figure S2 – Concentrations of stem non-structural carbohydrates (NSC_T), starch and soluble sugars in trees species with liana infestation ≥ 50% (grey) and in trees without liana infestation (white). For figure and analysis, we used the mean concentration per species and grouped both moist and dry site. Differences between groups were tested using Paired Samples Wilcoxon Test. Each box encompasses the 25th to 75th percentiles; the median is indicated by the horizontal line within each box while external horizontal lines indicate the 10th and 90th percentiles; dots indicate outliers.

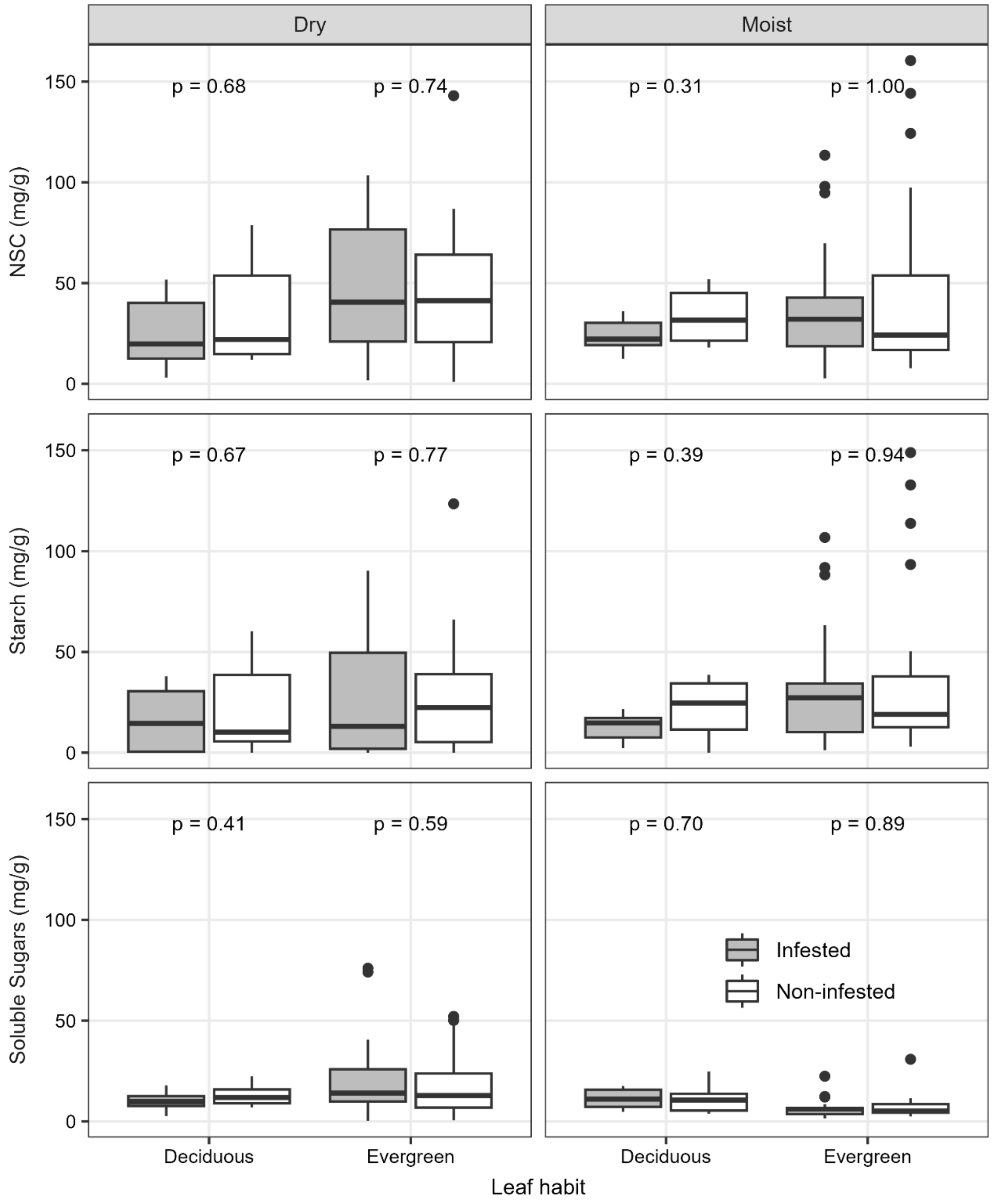

Figure S3 – Concentrations of stem non-structural carbohydrates (NSC), starch and soluble sugars in trees species with liana infestation ≥ 50% (grey) and in trees without liana infestation (white) separated per leaf habit. In the dry site only one species shows some level of deciduity, in the moist site we grouped a deciduous and a semi-deciduous species for analysis. For figure and analysis, we used the individual concentration of each tree. Differences between groups were tested using Wilcoxon Test. Each box encompasses the 25th to 75th percentiles; the median is indicated by the horizontal line within each box while external horizontal lines indicate the 10th and 90th percentiles; dots indicate outliers.

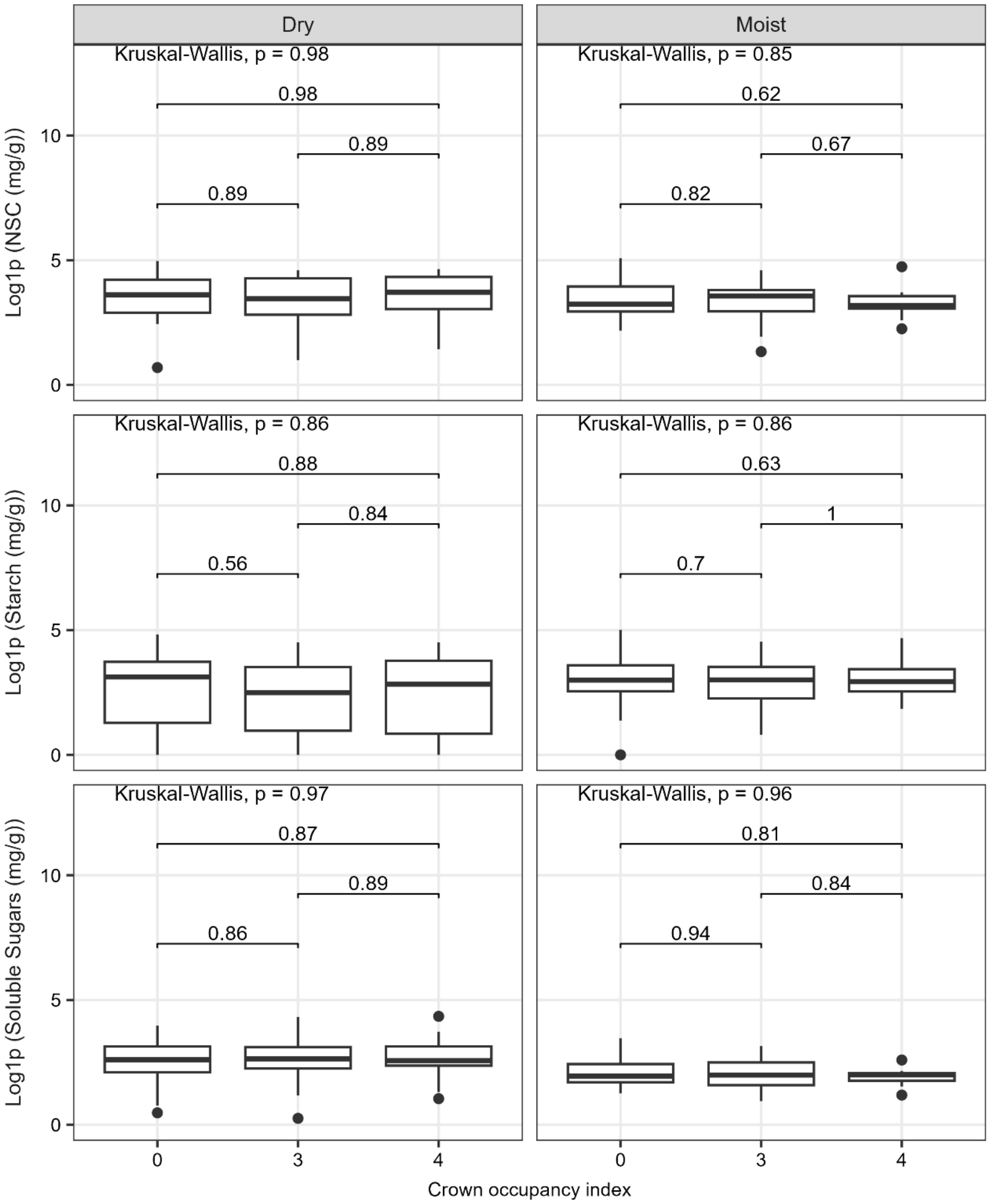

Figure S4– Comparison of stem non-structural carbohydrates (NSC), starch and soluble sugars concentration among distinct levels of Crown Occupancy Index (COI). COI = 0 represent those trees without liana infestation, COI = 3 or 4, meaning that liana cover was more than 50% or 75% of tree canopy cover, respectively. To test for differences in NSC concentrations among levels of infestation (COI) we used Kruskal-Wallis's rank sum test. Each box encompasses the 25th to 75th percentiles; the median is indicated by the horizontal line within each box while external horizontal lines indicate the 10th and 90th percentiles; dots indicate outliers.

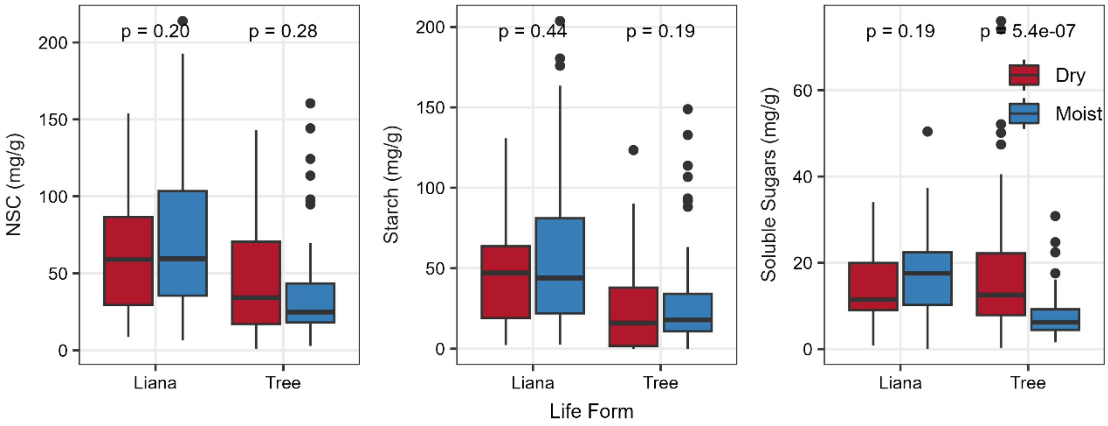

Figure S5 – Comparison of stem non-structural carbohydrates (NSC_T), starch and soluble sugars among sites for each life form. Differences between groups were tested using Wilcoxon rank sum test. Each box encompasses the 25th to 75th percentiles; the median is indicated by the horizontal line within each box while external horizontal lines indicate the 10th and 90th percentiles; dots indicate outliers.

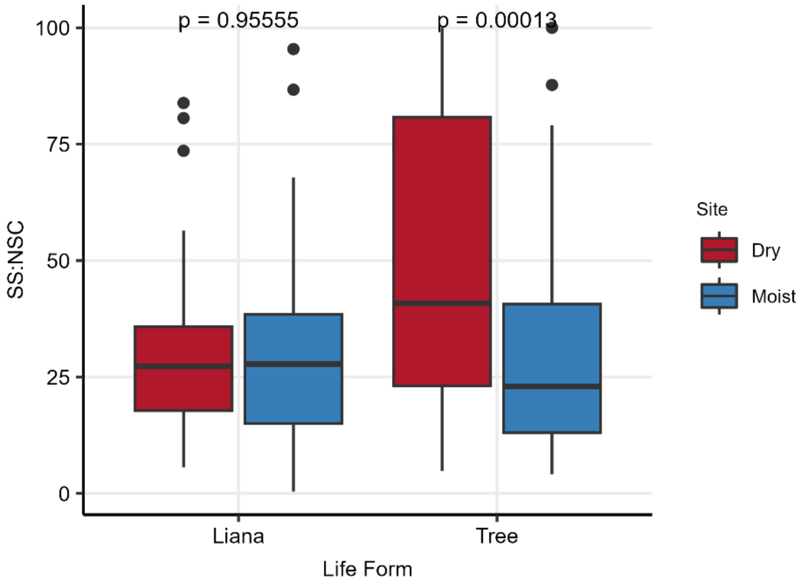

Figure S6 – Intersite comparison of stem proportion of non-structural carbohydrate (NSC) in the form of soluble sugars (SS:NSC) in lianas and trees. To test for differences between sites we used Wilcoxon rank sum test. Each box encompasses the 25th to 75th percentiles; the median is indicated by the horizontal line with each box while external horizontal lines indicate the 10th and 90th percentiles; dots indicate outliers.
